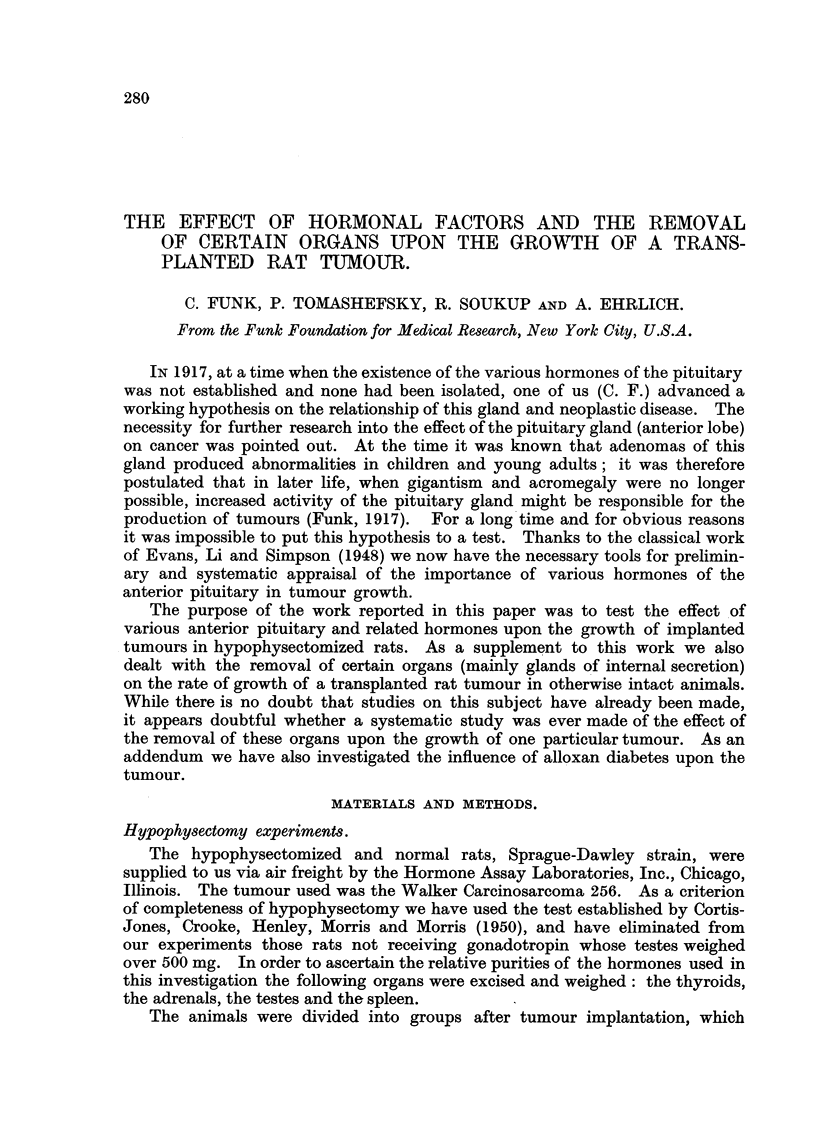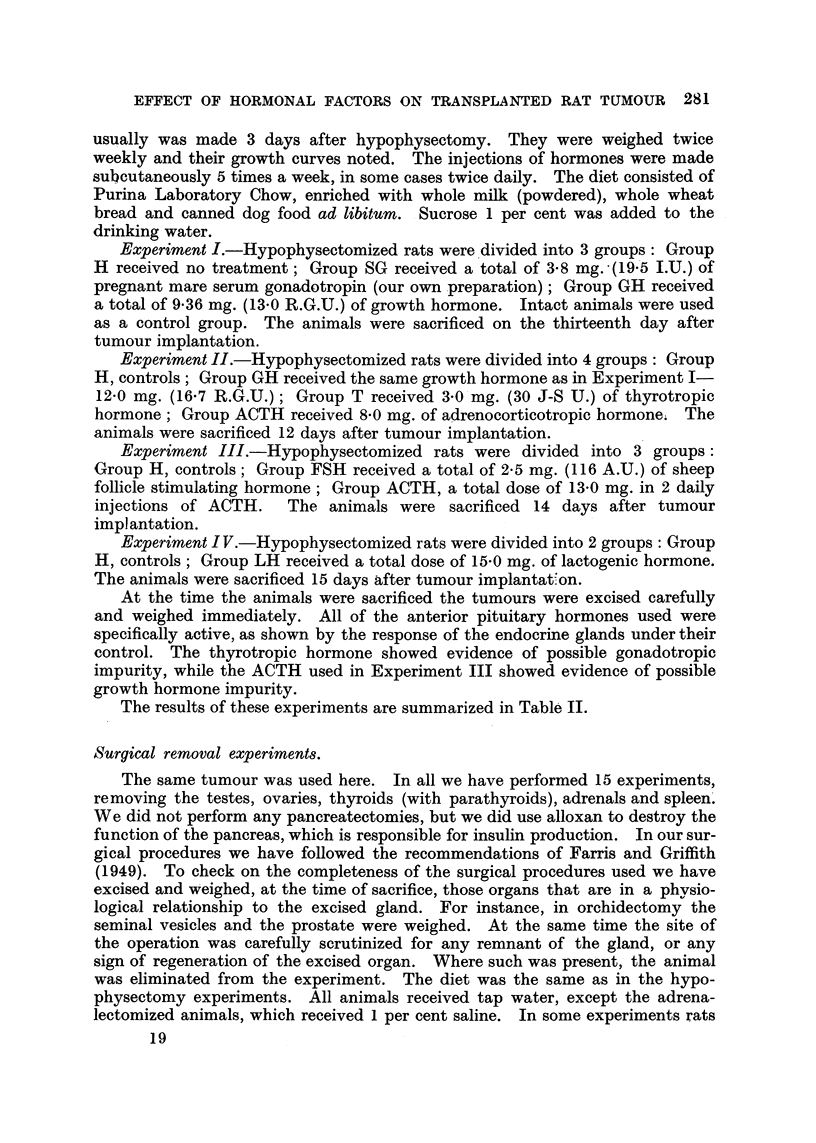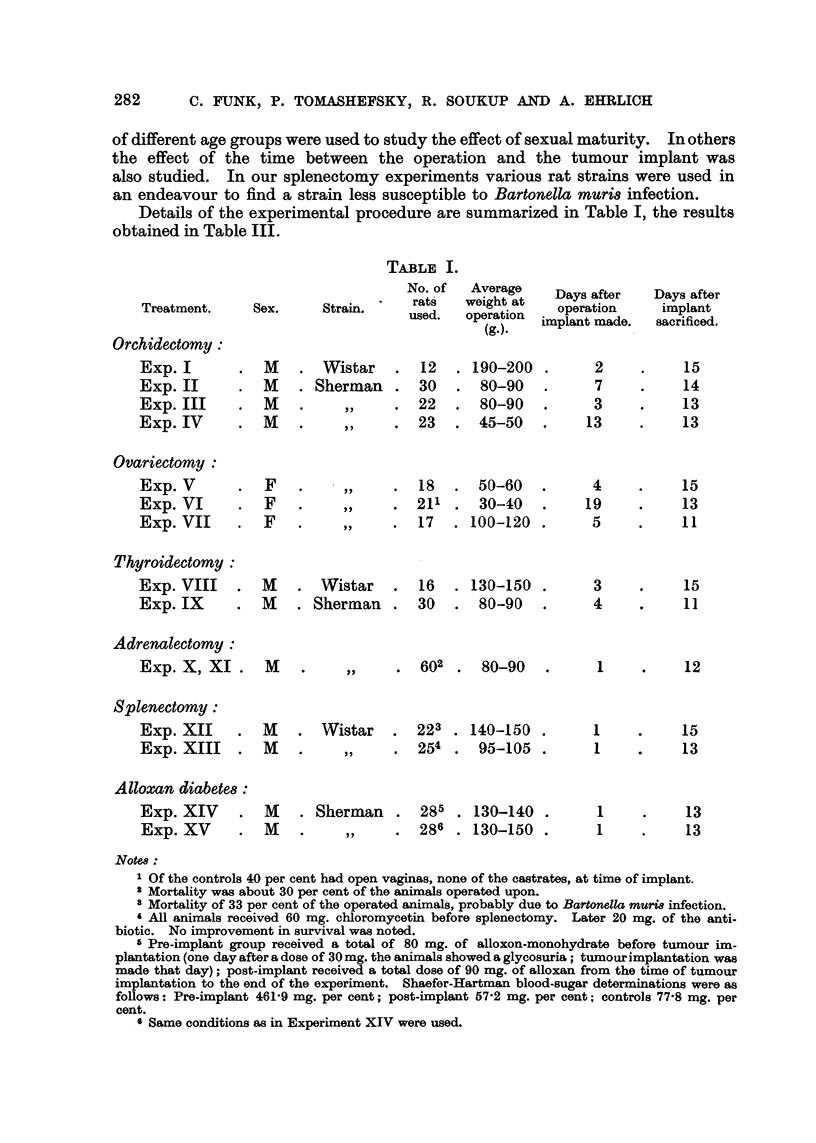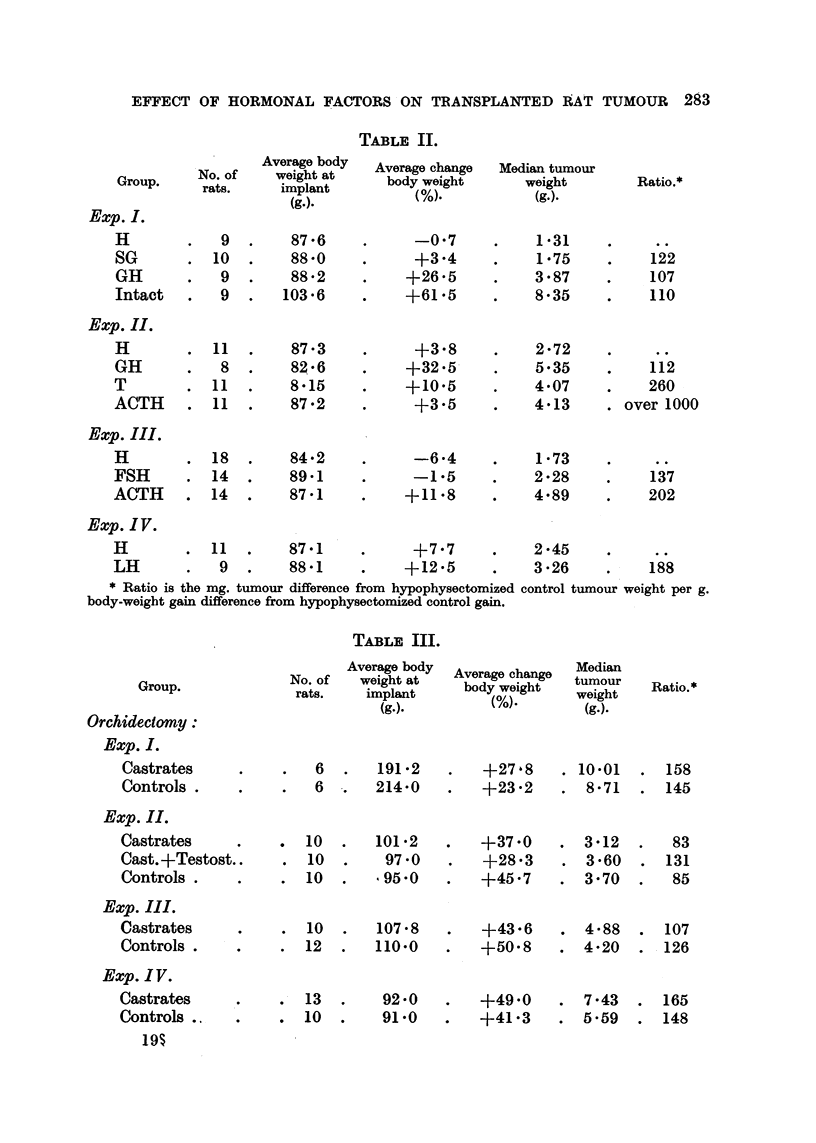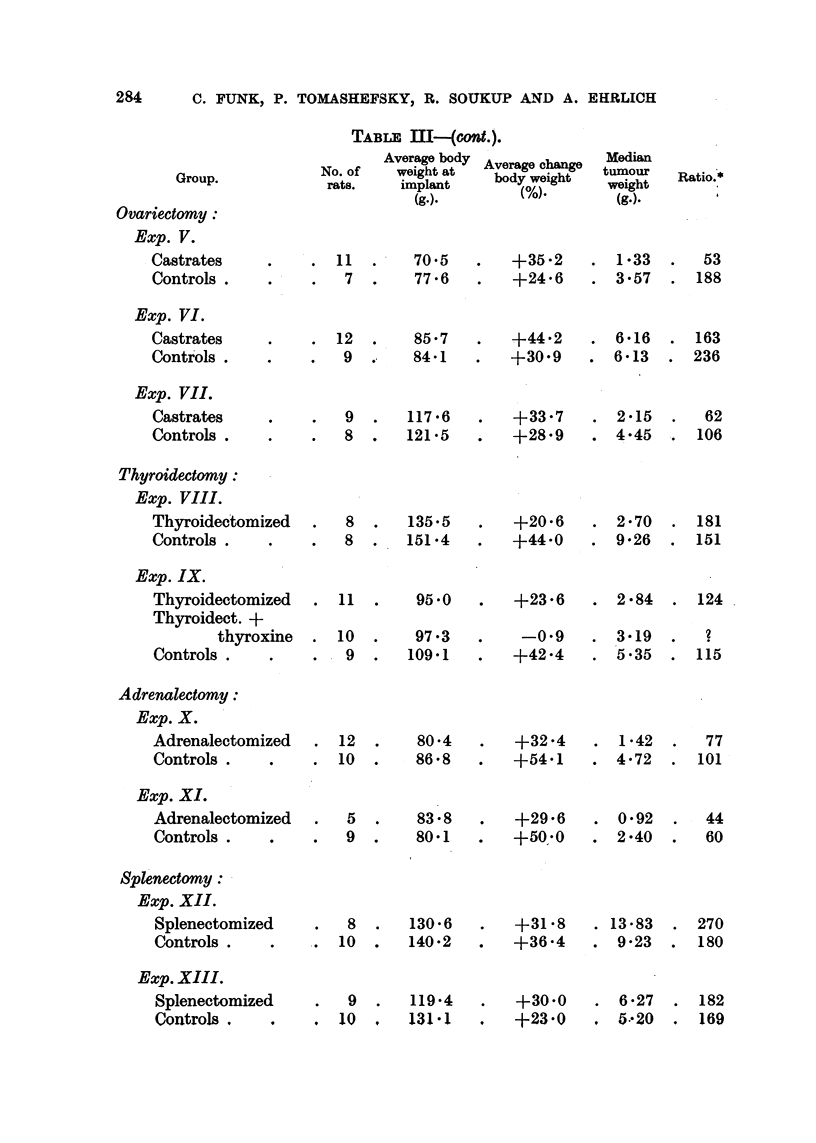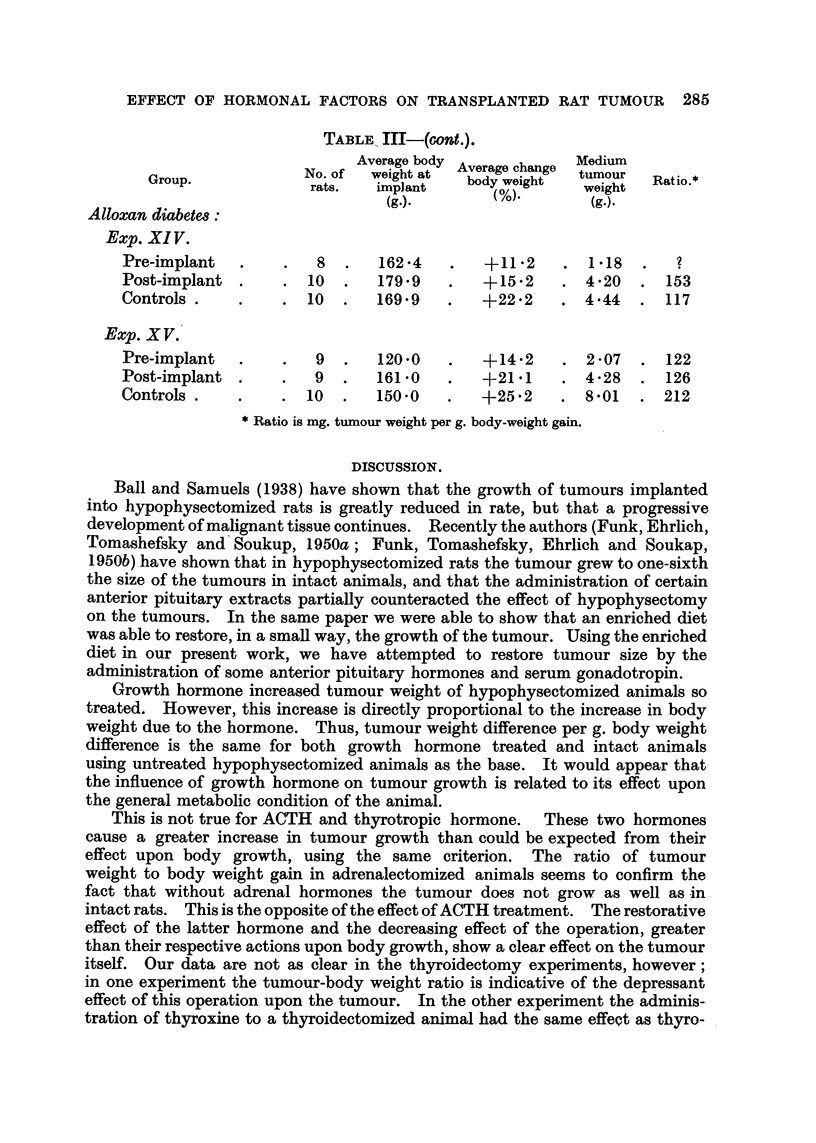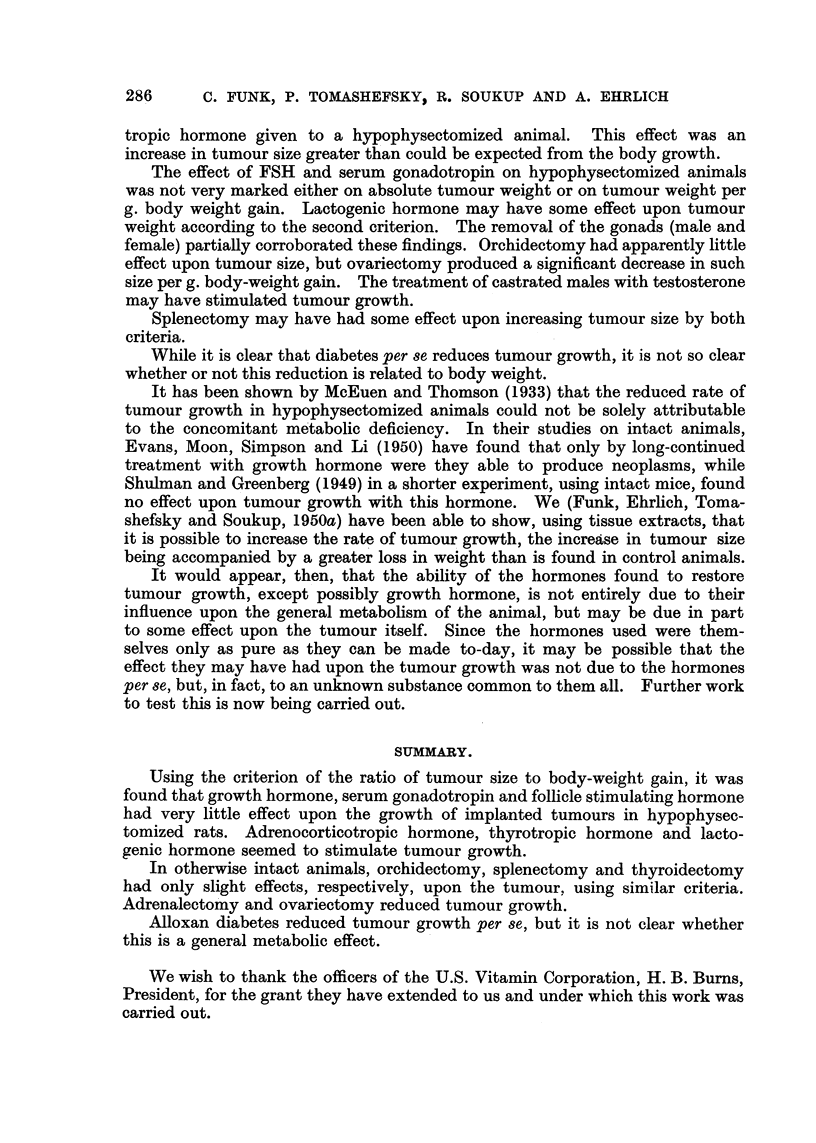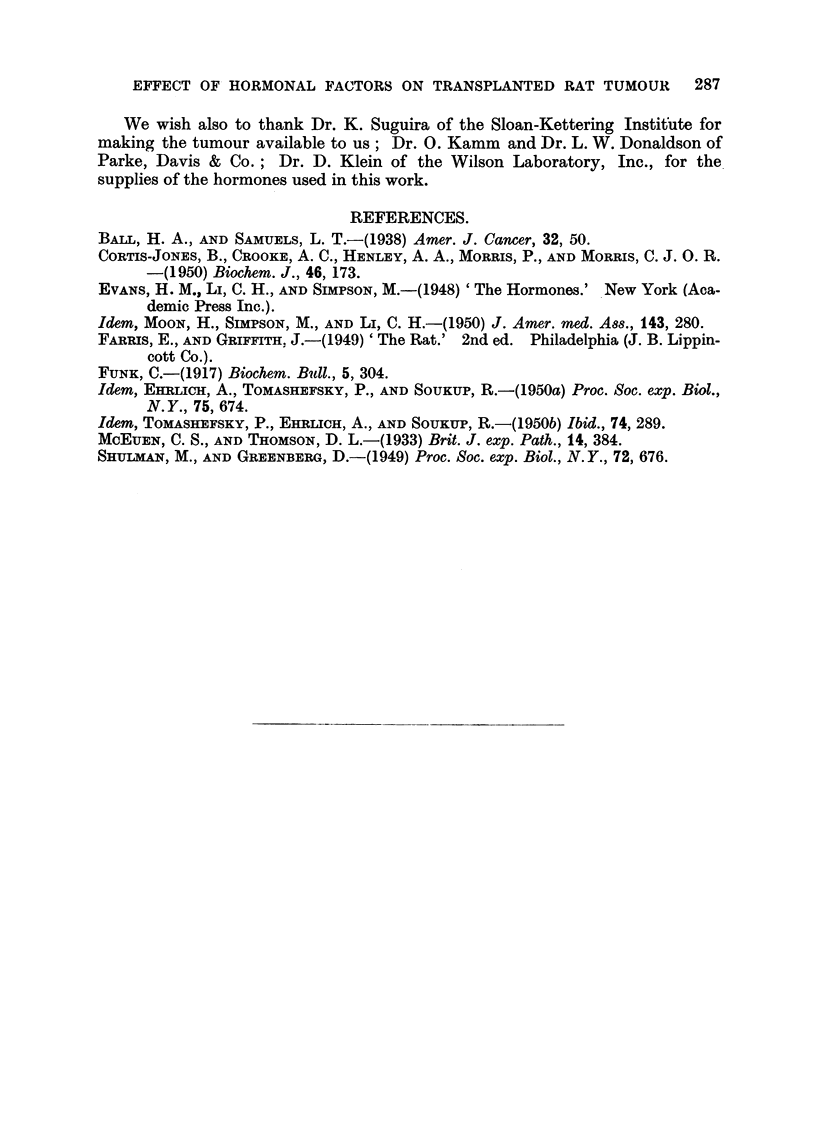# The Effect of Hormonal Factors and the Removal of Certain Organs upon the Growth of a Transplanted Rat Tumour

**DOI:** 10.1038/bjc.1951.29

**Published:** 1951-06

**Authors:** C. Funk, P. Tomashefsky, R. Soukup, A. Ehrlich


### THE EFFECT OF HORMONAL FACTORS AND THE REMOVAL OF CERTAIN ORGANS UPON THE GROWTH OF A TRANSPLANTED RAT TUMOUR.

C. FUNK, P. TOMASHEFSKY, R. SOUKUP AND A. EHRLICH.

*From the Funk Foundation for Medical Research, New York City, U.S.A.*

IN 1917, at a time when the existence of the various hormones of the pituitary was not established and none had been isolated, one of us (C. F.) advanced a working hypothesis on the relationship of this gland and neoplastic disease. The necessity for further research into the effect of the pituitary gland (anterior lobe) on cancer was pointed out. At the time it was known that adenomas of this gland produced abnormalities in children and young adults ; it was therefore postulated that in later life, when gigantism and acromegaly were no longer possible, increased activity of the pituitary gland might be responsible for the production of tumours (Funk, 1917). For a long time and for obvious reasons it was impossible to put this hypothesis to a test. Thanks to the classical work of Evans, Li and Simpson (1948) we now have the necessary tools for preliminary and systematic appraisal of the importance of various hormones of the anterior pituitary in tumour growth.

The purpose of the work reported in this paper was to test the effect of various anterior pituitary and related hormones upon the growth of implanted tumours in hypophysectomized rats. As a supplement to this work we also dealt with the removal of certain organs (mainly glands of internal secretion) on the rate of growth of a transplanted rat tumour in otherwise intact animals. While there is no doubt that studies on this subject have already been made, it appears doubtful whether a systematic study was ever made of the effect of the removal of these organs upon the growth of one particular tumour. As an addendum we have also investigated the influence of alloxan diabetes upon the tumour.

### MATERIALS AND METHODS.

### *Hypophysectomy experiments.*

The hypophysectomized and normal rats, Sprague-Dawley strain, were supplied to us via air freight by the Hormone Assay Laboratories, Inc., Chicago, Illinois. The tumour used was the Walker Carcinosarcoma 256. As a criterion of completeness of hypophysectomy we have used the test established by Cortis-Jones, Crooke, Henley, Morris and Morris (1950), and have eliminated from our experiments those rats not receiving gonadotropin whose testes weighed over 500 mg. In order to ascertain the relative purities of the hormones used in this investigation the following organs were excised and weighed : the thyroids, the adrenals, the testes and the spleen.

The animals were divided into groups after tumour implantation, which usually was made 3 days after hypophysectomy. They were weighed twice weekly and their growth curves noted. The injections of hormones were made subcutaneously 5 times a week, in some cases twice daily. The diet consisted of Purina Laboratory Chow, enriched with whole milk (powdered), whole wheat bread and canned dog food *ad libitum.* Sucrose 1 per cent was added to the drinking water.

*Experiment I.*—Hypophysectomized rats were divided into 3 groups : Group H received no treatment ; Group SG received a total of 3·8 mg. (19·5 I.U.) of pregnant mare serum gonadotropin (our own preparation) ; Group GH received a total of 9·36 mg. (13·0 R.G.U.) of growth hormone. Intact animals were used as a control group. The animals were sacrificed on the thirteenth day after tumour implantation.

*Experiment II.*—Hypophysectomized rats were divided into 4 groups : Group H, controls ; Group GH received the same growth hormone as in Experiment I—12·0 mg. (16·7 R.G.U.) ; Group T received 3·0 mg. (30 J-S U.) of thyrotropic hormone ; Group ACTH received 8·0 mg. of adrenocorticotropic hormone. The animals were sacrificed 12 days after tumour implantation.

*Experiment III.*—Hypophysectomized rats were divided into 3 groups : Group H, controls ; Group FSH received a total of 2·5 mg. (116 A.U.) of sheep follicle stimulating hormone ; Group ACTH, a total dose of 13·0 mg. in 2 daily injections of ACTH. The animals were sacrificed 14 days after tumour implantation.

*Experiment IV.*—Hypophysectomized rats were divided into 2 groups : Group H, controls ; Group LH received a total dose of 15·0 mg. of lactogenic hormone. The animals were sacrificed 15 days after tumour implantation.

At the time the animals were sacrificed the tumours were excised carefully and weighed immediately. All of the anterior pituitary hormones used were specifically active, as shown by the response of the endocrine glands under their control. The thyrotropic hormone showed evidence of possible gonadotropic impurity, while the ACTH used in Experiment III showed evidence of possible growth hormone impurity.

The results of these experiments are summarized in Table II.

*Surgical removal experiments.*

The same tumour was used here. In all we have performed 15 experiments, removing the testes, ovaries, thyroids (with parathyroids), adrenals and spleen. We did not perform any pancreatectomies, but we did use alloxan to destroy the function of the pancreas, which is responsible for insulin production. In our surgical procedures we have followed the recommendations of Farris and Griffith (1949). To check on the completeness of the surgical procedures used we have excised and weighed, at the time of sacrifice, those organs that are in a physiological relationship to the excised gland. For instance, in orchidectomy the seminal vesicles and the prostate were weighed. At the same time the site of the operation was carefully scrutinized for any remnant of the gland, or any sign of regeneration of the excised organ. Where such was present, the animal was eliminated from the experiment. The diet was the same as in the hypophysectomy experiments. All animals received tap water, except the adrenalectomized animals, which received 1 per cent saline. In some experiments rats of different age groups were used to study the effect of sexual maturity. In others the effect of the time between the operation and the tumour implant was also studied. In our splenectomy experiments various rat strains were used in an endeavour to find a strain less susceptible to *Bartonella muris* infection.

Details of the experimental procedure are summarized in Table I, the results obtained in Table III.

TABLE I.

| Treatment. | Sex. | Strain. | No. of rats used. | Average weight at operation (g.). | Days after operation implant made. | Days after implant sacrificed. |
|---|---|---|---|---|---|---|
| *Orchidectomy :* | | | | | | |
| Exp. I | M | Wistar | 12 | 190–200 | 2 | 15 |
| Exp. II | M | Sherman | 30 | 80–90 | 7 | 14 |
| Exp. III | M | ,, | 22 | 80–90 | 3 | 13 |
| Exp. IV | M | ,, | 23 | 45–50 | 13 | 13 |
| *Ovariectomy :* | | | | | | |
| Exp. V | F | ,, | 18 | 50–60 | 4 | 15 |
| Exp. VI | F | ,, | 21[1] | 30–40 | 19 | 13 |
| Exp. VII | F | ,, | 17 | 100–120 | 5 | 11 |
| *Thyroidectomy :* | | | | | | |
| Exp. VIII | M | Wistar | 16 | 130–150 | 3 | 15 |
| Exp. IX | M | Sherman | 30 | 80–90 | 4 | 11 |
| *Adrenalectomy :* | | | | | | |
| Exp. X, XI | M | ,, | 60[2] | 80–90 | 1 | 12 |
| *Splenectomy :* | | | | | | |
| Exp. XII | M | Wistar | 22[3] | 140–150 | 1 | 15 |
| Exp. XIII | M | ,, | 25[4] | 95–105 | 1 | 13 |
| *Alloxan diabetes :* | | | | | | |
| Exp. XIV | M | Sherman | 28[5] | 130–140 | 1 | 13 |
| Exp. XV | M | ,, | 28[6] | 130–150 | 1 | 13 |

*Notes :*

[1] Of the controls 40 per cent had open vaginas, none of the castrates, at time of implant.
[2] Mortality was about 30 per cent of the animals operated upon.
[3] Mortality of 33 per cent of the operated animals, probably due to *Bartonella muris* infection.
[4] All animals received 60 mg. chloromycetin before splenectomy. Later 20 mg. of the antibiotic. No improvement in survival was noted.
[5] Pre-implant group received a total of 80 mg. of alloxon-monohydrate before tumour implantation (one day after a dose of 30 mg. the animals showed a glycosuria ; tumour implantation was made that day); post-implant received a total dose of 90 mg. of alloxan from the time of tumour implantation to the end of the experiment. Shaefer-Hartman blood-sugar determinations were as follows: Pre-implant 461·9 mg. per cent; post-implant 57·2 mg. per cent; controls 77·8 mg. per cent.
[6] Same conditions as in Experiment XIV were used.

TABLE II.

| Group. | No. of rats. | Average body weight at implant (g.). | Average change body weight (%). | Median tumour weight (g.). | Ratio.* |
|---|---|---|---|---|---|
| *Exp. I.* | | | | | |
| H | 9 | 87·6 | −0·7 | 1·31 | .. |
| SG | 10 | 88·0 | +3·4 | 1·75 | 122 |
| GH | 9 | 88·2 | +26·5 | 3·87 | 107 |
| Intact | 9 | 103·6 | +61·5 | 8·35 | 110 |
| *Exp. II.* | | | | | |
| H | 11 | 87·3 | +3·8 | 2·72 | .. |
| GH | 8 | 82·6 | +32·5 | 5·35 | 112 |
| T | 11 | 8·15 | +10·5 | 4·07 | 260 |
| ACTH | 11 | 87·2 | +3·5 | 4·13 | over 1000 |
| *Exp. III.* | | | | | |
| H | 18 | 84·2 | −6·4 | 1·73 | .. |
| FSH | 14 | 89·1 | −1·5 | 2·28 | 137 |
| ACTH | 14 | 87·1 | +11·8 | 4·89 | 202 |
| *Exp. IV.* | | | | | |
| H | 11 | 87·1 | +7·7 | 2·45 | .. |
| LH | 9 | 88·1 | +12·5 | 3·26 | 188 |

* Ratio is the mg. tumour difference from hypophysectomized control tumour weight per g. body-weight gain difference from hypophysectomized control gain.

TABLE III.

| Group. | No. of rats. | Average body weight at implant (g.). | Average change body weight (%). | Median tumour weight (g.). | Ratio.* |
|---|---|---|---|---|---|
| *Orchidectomy :* | | | | | |
| *Exp. I.* | | | | | |
| Castrates | 6 | 191·2 | +27·8 | 10·01 | 158 |
| Controls | 6 | 214·0 | +23·2 | 8·71 | 145 |
| *Exp. II.* | | | | | |
| Castrates | 10 | 101·2 | +37·0 | 3·12 | 83 |
| Cast.+Testost. | 10 | 97·0 | +28·3 | 3·60 | 131 |
| Controls | 10 | 95·0 | +45·7 | 3·70 | 85 |
| *Exp. III.* | | | | | |
| Castrates | 10 | 107·8 | +43·6 | 4·88 | 107 |
| Controls | 12 | 110·0 | +50·8 | 4·20 | 126 |
| *Exp. IV.* | | | | | |
| Castrates | 13 | 92·0 | +49·0 | 7·43 | 165 |
| Controls | 10 | 91·0 | +41·3 | 5·59 | 148 |

TABLE III—(*cont.*).

| Group. | No. of rats. | Average body weight at implant (g.). | Average change body weight (%). | Median tumour weight (g.). | Ratio.* |
|---|---|---|---|---|---|
| *Ovariectomy :* | | | | | |
| *Exp. V.* | | | | | |
| Castrates | 11 | 70·5 | +35·2 | 1·33 | 53 |
| Controls | 7 | 77·6 | +24·6 | 3·57 | 188 |
| *Exp. VI.* | | | | | |
| Castrates | 12 | 85·7 | +44·2 | 6·16 | 163 |
| Controls | 9 | 84·1 | +30·9 | 6·13 | 236 |
| *Exp. VII.* | | | | | |
| Castrates | 9 | 117·6 | +33·7 | 2·15 | 62 |
| Controls | 8 | 121·5 | +28·9 | 4·45 | 106 |
| *Thyroidectomy :* | | | | | |
| *Exp. VIII.* | | | | | |
| Thyroidectomized | 8 | 135·5 | +20·6 | 2·70 | 181 |
| Controls | 8 | 151·4 | +44·0 | 9·26 | 151 |
| *Exp. IX.* | | | | | |
| Thyroidectomized | 11 | 95·0 | +23·6 | 2·84 | 124 |
| Thyroidect. + thyroxine | 10 | 97·3 | —0·9 | 3·19 | ? |
| Controls | 9 | 109·1 | +42·4 | 5·35 | 115 |
| *Adrenalectomy :* | | | | | |
| *Exp. X.* | | | | | |
| Adrenalectomized | 12 | 80·4 | +32·4 | 1·42 | 77 |
| Controls | 10 | 86·8 | +54·1 | 4·72 | 101 |
| *Exp. XI.* | | | | | |
| Adrenalectomized | 5 | 83·8 | +29·6 | 0·92 | 44 |
| Controls | 9 | 80·1 | +50·0 | 2·40 | 60 |
| *Splenectomy :* | | | | | |
| *Exp. XII.* | | | | | |
| Splenectomized | 8 | 130·6 | +31·8 | 13·83 | 270 |
| Controls | 10 | 140·2 | +36·4 | 9·23 | 180 |
| *Exp. XIII.* | | | | | |
| Splenectomized | 9 | 119·4 | +30·0 | 6·27 | 182 |
| Controls | 10 | 131·1 | +23·0 | 5·20 | 169 |

TABLE III—(*cont.*).

| Group. | No. of rats. | Average body weight at implant (g.). | Average change body weight (%). | Medium tumour weight (g.). | Ratio.* |
|---|---|---|---|---|---|
| *Alloxan diabetes :* | | | | | |
| *Exp. XIV.* | | | | | |
| Pre-implant | 8 | 162·4 | +11·2 | 1·18 | ? |
| Post-implant | 10 | 179·9 | +15·2 | 4·20 | 153 |
| Controls | 10 | 169·9 | +22·2 | 4·44 | 117 |
| *Exp. XV.* | | | | | |
| Pre-implant | 9 | 120·0 | +14·2 | 2·07 | 122 |
| Post-implant | 9 | 161·0 | +21·1 | 4·28 | 126 |
| Controls | 10 | 150·0 | +25·2 | 8·01 | 212 |

* Ratio is mg. tumour weight per g. body-weight gain.

### DISCUSSION.

Ball and Samuels (1938) have shown that the growth of tumours implanted into hypophysectomized rats is greatly reduced in rate, but that a progressive development of malignant tissue continues. Recently the authors (Funk, Ehrlich, Tomashefsky and Soukup, 1950*a* ; Funk, Tomashefsky, Ehrlich and Soukap, 1950*b*) have shown that in hypophysectomized rats the tumour grew to one-sixth the size of the tumours in intact animals, and that the administration of certain anterior pituitary extracts partially counteracted the effect of hypophysectomy on the tumours. In the same paper we were able to show that an enriched diet was able to restore, in a small way, the growth of the tumour. Using the enriched diet in our present work, we have attempted to restore tumour size by the administration of some anterior pituitary hormones and serum gonadotropin.

Growth hormone increased tumour weight of hypophysectomized animals so treated. However, this increase is directly proportional to the increase in body weight due to the hormone. Thus, tumour weight difference per g. body weight difference is the same for both growth hormone treated and intact animals using untreated hypophysectomized animals as the base. It would appear that the influence of growth hormone on tumour growth is related to its effect upon the general metabolic condition of the animal.

This is not true for ACTH and thyrotropic hormone. These two hormones cause a greater increase in tumour growth than could be expected from their effect upon body growth, using the same criterion. The ratio of tumour weight to body weight gain in adrenalectomized animals seems to confirm the fact that without adrenal hormones the tumour does not grow as well as in intact rats. This is the opposite of the effect of ACTH treatment. The restorative effect of the latter hormone and the decreasing effect of the operation, greater than their respective actions upon body growth, show a clear effect on the tumour itself. Our data are not as clear in the thyroidectomy experiments, however ; in one experiment the tumour-body weight ratio is indicative of the depressant effect of this operation upon the tumour. In the other experiment the administration of thyroxine to a thyroidectomized animal had the same effect as thyrotropic hormone given to a hypophysectomized animal. This effect was an increase in tumour size greater than could be expected from the body growth.

The effect of FSH and serum gonadotropin on hypophysectomized animals was not very marked either on absolute tumour weight or on tumour weight per g. body weight gain. Lactogenic hormone may have some effect upon tumour weight according to the second criterion. The removal of the gonads (male and female) partially corroborated these findings. Orchidectomy had apparently little effect upon tumour size, but ovariectomy produced a significant decrease in such size per g. body-weight gain. The treatment of castrated males with testosterone may have stimulated tumour growth.

Splenectomy may have had some effect upon increasing tumour size by both criteria.

While it is clear that diabetes *per se* reduces tumour growth, it is not so clear whether or not this reduction is related to body weight.

It has been shown by McEuen and Thomson (1933) that the reduced rate of tumour growth in hypophysectomized animals could not be solely attributable to the concomitant metabolic deficiency. In their studies on intact animals, Evans, Moon, Simpson and Li (1950) have found that only by long-continued treatment with growth hormone were they able to produce neoplasms, while Shulman and Greenberg (1949) in a shorter experiment, using intact mice, found no effect upon tumour growth with this hormone. We (Funk, Ehrlich, Tomashefsky and Soukup, 1950*a*) have been able to show, using tissue extracts, that it is possible to increase the rate of tumour growth, the increase in tumour size being accompanied by a greater loss in weight than is found in control animals.

It would appear, then, that the ability of the hormones found to restore tumour growth, except possibly growth hormone, is not entirely due to their influence upon the general metabolism of the animal, but may be due in part to some effect upon the tumour itself. Since the hormones used were themselves only as pure as they can be made to-day, it may be possible that the effect they may have had upon the tumour growth was not due to the hormones *per se*, but, in fact, to an unknown substance common to them all. Further work to test this is now being carried out.

### SUMMARY.

Using the criterion of the ratio of tumour size to body-weight gain, it was found that growth hormone, serum gonadotropin and follicle stimulating hormone had very little effect upon the growth of implanted tumours in hypophysectomized rats. Adrenocorticotropic hormone, thyrotropic hormone and lactogenic hormone seemed to stimulate tumour growth.

In otherwise intact animals, orchidectomy, splenectomy and thyroidectomy had only slight effects, respectively, upon the tumour, using similar criteria. Adrenalectomy and ovariectomy reduced tumour growth.

Alloxan diabetes reduced tumour growth *per se*, but it is not clear whether this is a general metabolic effect.

We wish to thank the officers of the U.S. Vitamin Corporation, H. B. Burns, President, for the grant they have extended to us and under which this work was carried out.

We wish also to thank Dr. K. Suguira of the Sloan-Kettering Institute for making the tumour available to us ; Dr. O. Kamm and Dr. L. W. Donaldson of Parke, Davis & Co. ; Dr. D. Klein of the Wilson Laboratory, Inc., for the supplies of the hormones used in this work.

### REFERENCES.

BALL, H. A., AND SAMUELS, L. T.—(1938) *Amer. J. Cancer*, **32**, 50.

CORTIS-JONES, B., CROOKE, A. C., HENLEY, A. A., MORRIS, P., AND MORRIS, C. J. O. R. —(1950) *Biochem. J.*, **46**, 173.

EVANS, H. M., LI, C. H., AND SIMPSON, M.—(1948) 'The Hormones.' New York (Academic Press Inc.).

*Idem*, MOON, H., SIMPSON, M., AND LI, C. H.—(1950) *J. Amer. med. Ass.*, **143**, 280.

FARRIS, E., AND GRIFFITH, J.—(1949) 'The Rat.' 2nd ed. Philadelphia (J. B. Lippincott Co.).

FUNK, C.—(1917) *Biochem. Bull.*, **5**, 304.

*Idem*, EHRLICH, A., TOMASHEFSKY, P., AND SOUKUP, R.—(1950*a*) *Proc. Soc. exp. Biol., N.Y.*, **75**, 674.

*Idem*, TOMASHEFSKY, P., EHRLICH, A., AND SOUKUP, R.—(1950*b*) *Ibid.*, **74**, 289.

MCEUEN, C. S., AND THOMSON, D. L.—(1933) *Brit. J. exp. Path.*, **14**, 384.

SHULMAN, M., AND GREENBERG, D.—(1949) *Proc. Soc. exp. Biol., N.Y.*, **72**, 676.